# Chlorido(2,3,7,8,12,13,17,18-octa­ethyl­porphyrinato)iron(III) dichloro­methane sesquisolvate

**DOI:** 10.1107/S1600536810020015

**Published:** 2010-06-05

**Authors:** Martin K. Safo, Kristin E. Buentello, Allen G. Oliver, W. Robert Scheidt

**Affiliations:** a Department of Chemistry and Biochemistry, University of Notre Dame, Notre Dame, IN 46556-5670, USA

## Abstract

The title mol­ecule, [Fe(C_36_H_44_N_4_)Cl]·1.5CH_2_Cl_2_, is a high-spin square-pyramidal iron(III) porphyrinate with an average value for the equatorial Fe—N bond lengths of 2.065 (3) Å and an axial Fe—Cl distance of 2.2430 (13) Å. The iron cation is displaced by 0.518 (1) Å from the 24-atom mean plane of the porphyrin ring. These values are typical for high-spin iron(III) porphyrinates.

## Related literature

For a review of porphyrinates, see: Scheidt (2000 ▶). Other crystalline phases containing the [Fe(OEP)Cl] moiety (OEP = octa­ethyl­porphyrin) have been reported by Ernst *et al.* (1977 ▶); Olmstead *et al.* (1999 ▶); Senge (2005 ▶). For synthetic details, see: Adler *et al.* (1970 ▶).
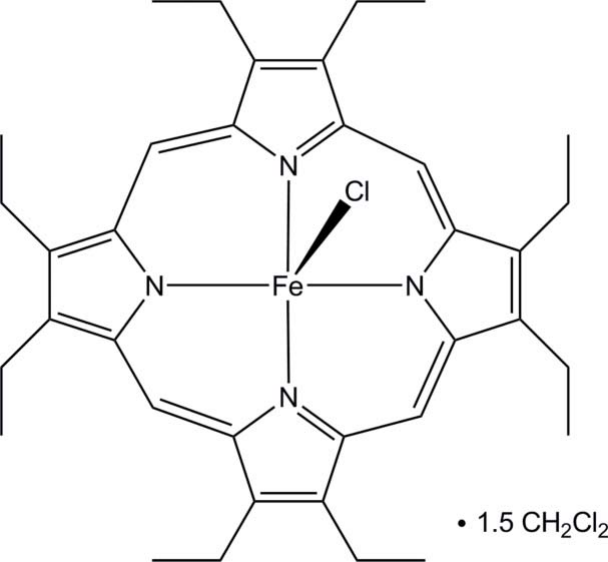


## Experimental

### 

#### Crystal data

[Fe(C_36_H_44_N_4_)Cl]·1.5CH_2_Cl_2_
*M* *_r_* = 751.44
Triclinic, 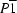

*a* = 10.062 (6) Å
*b* = 13.767 (5) Å
*c* = 14.754 (5) Å
α = 66.46 (2)°
β = 80.55 (2)°
γ = 76.10 (2)°
*V* = 1813.5 (14) Å^3^
*Z* = 2
Mo *K*α radiation
μ = 0.74 mm^−1^
*T* = 293 K
0.20 × 0.11 × 0.08 mm

#### Data collection

Enraf–Nonius FAST area-detector diffractometer
Absorption correction: multi-scan (*SADABS*; Sheldrick, 1995 ▶) *T*_min_ = 0.866, *T*_max_ = 0.943
9130 measured reflections
9130 independent reflections
7073 reflections with *I* > 2σ(*I*)
*R*_int_ = 0.060

#### Refinement

*R*[*F* ^2^ > 2σ(*F* ^2^)] = 0.059
*wR*(*F* ^2^) = 0.148
*S* = 1.05
9130 reflections
441 parameters
H-atom parameters constrained
Δρ_max_ = 1.02 e Å^−3^
Δρ_min_ = −0.69 e Å^−3^

### 

Data collection: *MADNES* (Messerschmidt & Pflugrath, 1987 ▶); cell refinement: *MADNES*; data reduction: *MADNES*; program(s) used to solve structure: *SHELXS97* (Sheldrick, 2008 ▶); program(s) used to refine structure: *SHELXL97* (Sheldrick, 2008 ▶); molecular graphics: *ORTEPII* (Johnson, 1976 ▶); software used to prepare material for publication: *SHELXL97* and *publCIF* (Westrip, 2010 ▶).

## Supplementary Material

Crystal structure: contains datablocks I, global. DOI: 10.1107/S1600536810020015/wm2352sup1.cif

Structure factors: contains datablocks I. DOI: 10.1107/S1600536810020015/wm2352Isup2.hkl

Additional supplementary materials:  crystallographic information; 3D view; checkCIF report

## Figures and Tables

**Table:** 

Fe—N2	2.060 (3)
Fe—N4	2.066 (3)
Fe—N1	2.066 (2)
Fe—N3	2.067 (2)
Fe—Cl1	2.2430 (13)

**Table:** 

N2—Fe—N4	154.50 (10)
N1—Fe—N3	153.12 (10)
N2—Fe—Cl1	102.63 (8)
N4—Fe—Cl1	102.87 (8)
N1—Fe—Cl1	103.92 (7)
N3—Fe—Cl1	102.96 (7)

**Fig. 1.**
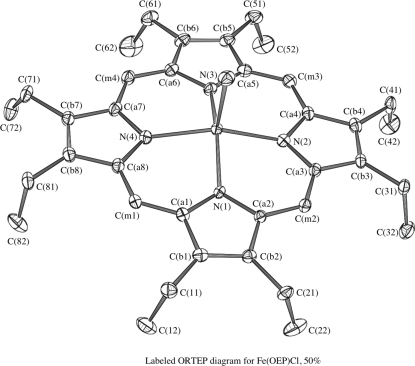
: ORTEP diagram for [Fe(OEP)Cl] with atom labels and displacement ellipsoids at the 50% probability level. Methylene chloride solvent molecules and hydrogen atoms have been removed for clarity.

## supplementary crystallographic information

### Comment

The title compound (in the reported crystalline form) has been used for many years in the principal author's laboratory as a convenient starting material for many studies of porphyrin derivatives. The square-pyramidal coordination of the central irn(III) atom, with an average equatorial Fe—N distance of 2.065 (3) Å and 2.2430 (13) Å for the axial Fe—Cl distance, is typical for high-spin chloride derivatives (Scheidt, 2000). The iron atom is displaced by 0.518 (1) Å from the 24 atom mean plane and 0.468 (1) Å from the plane of the four nitrogen atoms. The core has a modest saddled conformation. The conformation of the the molecule with its eight peripheral ethyl groups is unusual with all eight groups pointing away from the axial chloride ligand (see Fig. 1), resulting in a molecule with a spider-like shape. This geometry leads to well-separated iron atoms with the closest Fe···Fe separation of 9.711 (3) Å that is larger than typical for OEP (OEP = octaethylporphyrin) derivatives.

Three different crystalline species containing the [Fe(OEP)Cl] moiety have been previously reported by Ernst *et al.* (1977), Olmstead *et al.* (1999) and Senge (2005). In comparison with the current derivative they have a different conformation of the peripheral ethyl groups.

### Experimental

Iron(II) chloride was purchased from Fisher and H_2_OEP from Midcentury Chemicals. [Fe(OEP)Cl] was prepared by reaction of iron(II) chloride in dimethyl formamide as described by Adler *et al.* (1970)). Single crystals were obtained by slow evaporation of methylene chloride solutions.

### Refinement

The H atoms attached to C atoms of the porphyrin ring were positioned geometrically and allowed to ride on their parent atoms, with a C—H distance of 0.93 Å and *U*_iso_(H) = 1.2*U*_eq_(C). Methylene and methyl H atoms were likewise positioned geometrically and refined as riding atoms, with C—H = 0.97 Å (methylene) and C—H = 0.96 Å (methyl) and *U*_iso_(H) = 1.2*U*_eq_(C).

One of the methylene chloride molecules of crystallization is disordered across the inversion center at [0.5, 0, 1] and has been modelled with half occupancy atoms. The other methylene chloride occupies a general position in the lattice and was modelled at full occupancy.

### Crystal data

**Table:**

[Fe(C_36_H_44_N_4_)Cl]·1.5CH_2_Cl_2_	*Z* = 2
*M**_r_* = 751.44	*F*(000) = 788
Triclinic, *P*1	*D*_x_ = 1.376 Mg m^−^^3^
Hall symbol: -P 1	Mo *K*α radiation, λ = 0.71073 Å
*a* = 10.062 (6) Å	Cell parameters from 250 reflections
*b* = 13.767 (5) Å	θ = 2.5–26.0°
*c* = 14.754 (5) Å	µ = 0.74 mm^−^^1^
α = 66.46 (2)°	*T* = 293 K
β = 80.55 (2)°	Needle, dark purple
γ = 76.10 (2)°	0.20 × 0.11 × 0.08 mm
*V* = 1813.5 (14) Å^3^	

### Data collection

**Table:**

Enraf–Nonius FAST area-detector diffractometer	9130 independent reflections
Radiation source: rotating anode X-ray tube	7073 reflections with *I* > 2σ(*I*)
graphite	*R*_int_ = 0.060
Detector resolution: 9.23 pixels mm^-1^	θ_max_ = 29.8°, θ_min_ = 2.4°
Ellipsoid–mask fitting scans	*h* = −13→14
Absorption correction: multi-scan (*SADABS*; Sheldrick, 1995)	*k* = −16→19
*T*_min_ = 0.866, *T*_max_ = 0.943	*l* = 0→20
9130 measured reflections	

### Refinement

**Table:**

Refinement on *F*^2^	Primary atom site location: structure-invariant direct methods
Least-squares matrix: full	Secondary atom site location: difference Fourier map
*R*[*F*^2^ > 2σ(*F*^2^)] = 0.059	Hydrogen site location: inferred from neighbouring sites
*wR*(*F*^2^) = 0.148	H-atom parameters constrained
*S* = 1.05	*w* = 1/[σ^2^(*F*_o_^2^) + (0.0448*P*)^2^ + 5.0201*P*] where *P* = (*F*_o_^2^ + 2*F*_c_^2^)/3
9130 reflections	(Δ/σ)_max_ = 0.001
441 parameters	Δρ_max_ = 1.02 e Å^−^^3^
0 restraints	Δρ_min_ = −0.68 e Å^−^^3^

### Special details

**Table:**

Experimental. Diffraction data were measured with an Enraf Nonius FAST area detector to 59.54 deg in 2 theta. With the hardware and software supplied for the diffractometer, the data collection process provides substantial redundancy but not necessarily completion up to the limiting resolution. At a resolution of 0.83 Å (52 deg in 2 theta) essentially full coverage of data were met. Successful and suitable refinement of the structure supports this.
Geometry. All esds (except the esd in the dihedral angle between two l.s. planes) are estimated using the full covariance matrix. The cell esds are taken into account individually in the estimation of esds in distances, angles and torsion angles; correlations between esds in cell parameters are only used when they are defined by crystal symmetry. An approximate (isotropic) treatment of cell esds is used for estimating esds involving l.s. planes.
Refinement. Refinement of *F*^2^ against ALL reflections. The weighted *R*-factor wR and goodness of fit *S* are based on *F*^2^, conventional *R*-factors *R* are based on *F*, with *F* set to zero for negative *F*^2^. The threshold expression of *F*^2^ > σ(*F*^2^) is used only for calculating *R*-factors(gt) etc. and is not relevant to the choice of reflections for refinement. *R*-factors based on *F*^2^ are statistically about twice as large as those based on *F*, and *R*- factors based on ALL data will be even larger.

### Fractional atomic coordinates and isotropic or equivalent isotropic displacement parameters (Å^2^)

**Table:**

	*x*	*y*	*z*	*U*_iso_*/*U*_eq_	Occ. (<1)
Fe	0.25435 (4)	0.37510 (3)	0.32355 (3)	0.01287 (10)	
Cl1	0.08498 (7)	0.28040 (6)	0.37840 (6)	0.02095 (15)	
N1	0.3046 (2)	0.38438 (18)	0.44976 (17)	0.0134 (4)	
N2	0.1490 (2)	0.53203 (19)	0.28837 (18)	0.0153 (5)	
N3	0.2765 (2)	0.40653 (19)	0.17326 (17)	0.0155 (5)	
N4	0.4286 (2)	0.25643 (19)	0.33618 (18)	0.0157 (5)	
CA1	0.3884 (3)	0.3059 (2)	0.5196 (2)	0.0148 (5)	
CA2	0.2377 (3)	0.4580 (2)	0.4922 (2)	0.0138 (5)	
CA3	0.1014 (3)	0.5843 (2)	0.3531 (2)	0.0145 (5)	
CA4	0.0834 (3)	0.5949 (2)	0.2033 (2)	0.0157 (5)	
CA5	0.1976 (3)	0.4875 (2)	0.1020 (2)	0.0166 (5)	
CA6	0.3542 (3)	0.3396 (2)	0.1268 (2)	0.0173 (5)	
CA7	0.4799 (3)	0.2059 (2)	0.2702 (2)	0.0168 (5)	
CA8	0.4961 (3)	0.1942 (2)	0.4208 (2)	0.0158 (5)	
CB1	0.3752 (3)	0.3307 (2)	0.6076 (2)	0.0158 (5)	
CB2	0.2824 (3)	0.4256 (2)	0.5899 (2)	0.0147 (5)	
CB3	0.0033 (3)	0.6824 (2)	0.3080 (2)	0.0148 (5)	
CB4	−0.0072 (3)	0.6885 (2)	0.2142 (2)	0.0179 (6)	
CB5	0.2272 (3)	0.4728 (2)	0.0086 (2)	0.0191 (6)	
CB6	0.3255 (3)	0.3818 (2)	0.0240 (2)	0.0200 (6)	
CB7	0.5800 (3)	0.1091 (2)	0.3153 (2)	0.0170 (5)	
CB8	0.5911 (3)	0.1035 (2)	0.4079 (2)	0.0174 (5)	
C11	0.4545 (3)	0.2652 (3)	0.6968 (2)	0.0203 (6)	
H11A	0.4625	0.1891	0.7105	0.024*	
H11B	0.4049	0.2795	0.7537	0.024*	
C21	0.2368 (3)	0.4896 (2)	0.6554 (2)	0.0181 (6)	
H21A	0.2517	0.4420	0.7237	0.022*	
H21B	0.1392	0.5190	0.6514	0.022*	
C31	−0.0673 (3)	0.7605 (2)	0.3557 (2)	0.0184 (6)	
H31A	−0.0960	0.7204	0.4240	0.022*	
H31B	−0.1492	0.8032	0.3219	0.022*	
C41	−0.0905 (3)	0.7768 (2)	0.1356 (2)	0.0215 (6)	
H41A	−0.1684	0.8123	0.1667	0.026*	
H41B	−0.1256	0.7457	0.0981	0.026*	
C51	0.1646 (4)	0.5492 (3)	−0.0860 (2)	0.0246 (6)	
H51A	0.0683	0.5753	−0.0714	0.030*	
H51B	0.1703	0.5109	−0.1296	0.030*	
C61	0.4006 (4)	0.3357 (3)	−0.0506 (2)	0.0261 (7)	
H61A	0.3454	0.3607	−0.1066	0.031*	
H61B	0.4126	0.2576	−0.0209	0.031*	
C71	0.6548 (3)	0.0321 (2)	0.2667 (2)	0.0215 (6)	
H71A	0.5918	0.0242	0.2280	0.026*	
H71B	0.6832	−0.0380	0.3179	0.026*	
C81	0.6882 (3)	0.0236 (2)	0.4824 (2)	0.0218 (6)	
H81A	0.7153	−0.0426	0.4698	0.026*	
H81B	0.6417	0.0068	0.5483	0.026*	
C12	0.5981 (3)	0.2906 (3)	0.6824 (3)	0.0300 (7)	
H12A	0.6459	0.2466	0.7409	0.045*	
H12B	0.5906	0.3654	0.6707	0.045*	
H12C	0.6480	0.2757	0.6266	0.045*	
C22	0.3140 (4)	0.5818 (3)	0.6261 (3)	0.0337 (8)	
H22A	0.2797	0.6222	0.6679	0.051*	
H22B	0.3009	0.6284	0.5582	0.051*	
H22C	0.4102	0.5528	0.6336	0.051*	
C32	0.0221 (3)	0.8363 (3)	0.3540 (3)	0.0260 (7)	
H32A	−0.0304	0.8866	0.3826	0.039*	
H32B	0.0530	0.8751	0.2867	0.039*	
H32C	0.1001	0.7951	0.3915	0.039*	
C42	−0.0075 (4)	0.8600 (3)	0.0652 (3)	0.0347 (8)	
H42A	−0.0640	0.9139	0.0148	0.052*	
H42B	0.0704	0.8251	0.0348	0.052*	
H42C	0.0236	0.8937	0.1015	0.052*	
C52	0.2359 (5)	0.6451 (3)	−0.1389 (3)	0.0359 (8)	
H52A	0.1908	0.6931	−0.1977	0.054*	
H52B	0.3301	0.6200	−0.1567	0.054*	
H52C	0.2316	0.6828	−0.0958	0.054*	
C62	0.5393 (4)	0.3664 (4)	−0.0875 (3)	0.0462 (11)	
H62A	0.5847	0.3317	−0.1323	0.069*	
H62B	0.5937	0.3437	−0.0323	0.069*	
H62C	0.5277	0.4433	−0.1214	0.069*	
C72	0.7802 (4)	0.0664 (3)	0.1995 (3)	0.0315 (8)	
H72A	0.8264	0.0109	0.1753	0.047*	
H72B	0.8414	0.0780	0.2362	0.047*	
H72C	0.7523	0.1321	0.1446	0.047*	
C82	0.8159 (3)	0.0668 (3)	0.4785 (3)	0.0318 (8)	
H82A	0.8786	0.0117	0.5237	0.048*	
H82B	0.7902	0.1286	0.4970	0.048*	
H82C	0.8594	0.0873	0.4125	0.048*	
CM1	0.4754 (3)	0.2172 (2)	0.5060 (2)	0.0155 (5)	
HM1	0.5249	0.1682	0.5593	0.019*	
CM2	0.1418 (3)	0.5494 (2)	0.4479 (2)	0.0155 (5)	
HM2	0.1009	0.5911	0.4852	0.019*	
CM3	0.1055 (3)	0.5733 (2)	0.1173 (2)	0.0180 (5)	
HM3	0.0534	0.6209	0.0649	0.022*	
CM4	0.4449 (3)	0.2450 (2)	0.1729 (2)	0.0189 (6)	
HM4	0.4863	0.2038	0.1353	0.023*	
CS1	0.1683 (4)	0.1052 (3)	0.2470 (3)	0.0346 (8)	
HS1A	0.0914	0.0780	0.2910	0.042*	
HS1B	0.2004	0.1496	0.2726	0.042*	
Cl2	0.30036 (11)	−0.00366 (8)	0.24654 (9)	0.0445 (2)	
Cl3	0.11326 (13)	0.18428 (11)	0.12904 (9)	0.0579 (3)	
CS2	0.5781 (11)	−0.0403 (7)	1.0177 (9)	0.052 (3)	0.50
HS2A	0.5710	−0.0752	1.0895	0.063*	0.50
HS2B	0.6417	−0.0895	0.9913	0.063*	0.50
Cl4	0.6407 (3)	0.0771 (2)	0.9837 (2)	0.0636 (7)	0.50
Cl5	0.4123 (3)	−0.0124 (4)	0.9724 (3)	0.0754 (9)	0.50

### Atomic displacement parameters (Å^2^)

**Table:**

	*U*^11^	*U*^22^	*U*^33^	*U*^12^	*U*^13^	*U*^23^
Fe	0.01256 (18)	0.01266 (19)	0.01360 (19)	−0.00042 (14)	−0.00143 (14)	−0.00611 (14)
Cl1	0.0188 (3)	0.0219 (3)	0.0255 (4)	−0.0070 (3)	0.0022 (3)	−0.0121 (3)
N1	0.0138 (10)	0.0130 (10)	0.0146 (11)	0.0001 (8)	−0.0010 (9)	−0.0079 (9)
N2	0.0156 (11)	0.0150 (11)	0.0158 (11)	−0.0025 (9)	−0.0014 (9)	−0.0065 (9)
N3	0.0176 (11)	0.0171 (11)	0.0129 (11)	−0.0015 (9)	−0.0006 (9)	−0.0080 (9)
N4	0.0143 (11)	0.0163 (11)	0.0170 (12)	−0.0008 (9)	−0.0013 (9)	−0.0080 (9)
CA1	0.0139 (12)	0.0146 (12)	0.0145 (13)	−0.0016 (10)	−0.0016 (10)	−0.0046 (10)
CA2	0.0104 (11)	0.0151 (12)	0.0178 (13)	−0.0021 (10)	0.0004 (10)	−0.0090 (10)
CA3	0.0123 (12)	0.0140 (12)	0.0176 (13)	−0.0024 (10)	0.0016 (10)	−0.0075 (10)
CA4	0.0138 (12)	0.0131 (12)	0.0181 (13)	−0.0009 (10)	−0.0015 (10)	−0.0044 (10)
CA5	0.0181 (13)	0.0180 (13)	0.0136 (13)	−0.0041 (11)	−0.0017 (10)	−0.0052 (11)
CA6	0.0187 (13)	0.0179 (13)	0.0166 (13)	−0.0037 (11)	0.0000 (11)	−0.0083 (11)
CA7	0.0151 (12)	0.0169 (13)	0.0199 (14)	−0.0025 (10)	0.0025 (10)	−0.0103 (11)
CA8	0.0141 (12)	0.0124 (12)	0.0197 (14)	−0.0016 (10)	−0.0008 (10)	−0.0054 (10)
CB1	0.0158 (13)	0.0178 (13)	0.0149 (13)	−0.0055 (10)	0.0002 (10)	−0.0065 (11)
CB2	0.0146 (12)	0.0170 (13)	0.0136 (13)	−0.0042 (10)	0.0012 (10)	−0.0071 (10)
CB3	0.0110 (11)	0.0118 (12)	0.0198 (14)	−0.0005 (10)	0.0004 (10)	−0.0059 (10)
CB4	0.0150 (13)	0.0140 (13)	0.0210 (14)	−0.0009 (10)	−0.0025 (11)	−0.0036 (11)
CB5	0.0239 (14)	0.0194 (14)	0.0154 (13)	−0.0055 (11)	−0.0024 (11)	−0.0069 (11)
CB6	0.0259 (15)	0.0214 (14)	0.0161 (14)	−0.0052 (12)	−0.0004 (11)	−0.0106 (11)
CB7	0.0142 (12)	0.0131 (12)	0.0229 (14)	−0.0022 (10)	−0.0007 (11)	−0.0064 (11)
CB8	0.0140 (12)	0.0149 (13)	0.0213 (14)	−0.0011 (10)	−0.0016 (11)	−0.0057 (11)
C11	0.0222 (14)	0.0226 (14)	0.0147 (13)	−0.0006 (12)	−0.0038 (11)	−0.0069 (11)
C21	0.0237 (14)	0.0184 (13)	0.0148 (13)	−0.0036 (11)	−0.0011 (11)	−0.0093 (11)
C31	0.0159 (13)	0.0154 (13)	0.0238 (15)	0.0010 (10)	−0.0004 (11)	−0.0101 (11)
C41	0.0233 (15)	0.0140 (13)	0.0212 (15)	0.0066 (11)	−0.0043 (12)	−0.0052 (11)
C51	0.0324 (17)	0.0251 (15)	0.0182 (15)	−0.0052 (13)	−0.0075 (13)	−0.0081 (12)
C61	0.0331 (17)	0.0305 (17)	0.0199 (15)	−0.0029 (14)	−0.0015 (13)	−0.0168 (13)
C71	0.0218 (14)	0.0166 (14)	0.0268 (16)	0.0012 (11)	0.0011 (12)	−0.0129 (12)
C81	0.0173 (13)	0.0182 (14)	0.0267 (16)	0.0049 (11)	−0.0041 (12)	−0.0091 (12)
C12	0.0213 (15)	0.040 (2)	0.0289 (18)	−0.0022 (14)	−0.0101 (13)	−0.0128 (15)
C22	0.044 (2)	0.0336 (19)	0.036 (2)	−0.0178 (16)	0.0046 (16)	−0.0229 (16)
C32	0.0262 (16)	0.0179 (14)	0.0363 (18)	−0.0034 (12)	0.0001 (14)	−0.0143 (13)
C42	0.049 (2)	0.0223 (16)	0.0245 (17)	−0.0078 (15)	−0.0041 (16)	0.0009 (13)
C52	0.056 (2)	0.0276 (18)	0.0210 (17)	−0.0121 (17)	−0.0078 (16)	−0.0014 (14)
C62	0.042 (2)	0.065 (3)	0.041 (2)	−0.016 (2)	0.0152 (19)	−0.034 (2)
C72	0.0282 (17)	0.0280 (17)	0.0352 (19)	0.0034 (14)	0.0086 (14)	−0.0181 (15)
C82	0.0174 (15)	0.043 (2)	0.0323 (19)	−0.0021 (14)	−0.0074 (13)	−0.0120 (16)
CM1	0.0132 (12)	0.0149 (12)	0.0172 (13)	0.0005 (10)	−0.0034 (10)	−0.0058 (10)
CM2	0.0142 (12)	0.0156 (13)	0.0179 (13)	−0.0009 (10)	0.0001 (10)	−0.0092 (11)
CM3	0.0186 (13)	0.0173 (13)	0.0173 (14)	−0.0016 (11)	−0.0042 (11)	−0.0057 (11)
CM4	0.0171 (13)	0.0188 (14)	0.0220 (14)	−0.0019 (11)	0.0008 (11)	−0.0109 (12)
CS1	0.0310 (18)	0.039 (2)	0.036 (2)	−0.0044 (16)	0.0009 (15)	−0.0191 (17)
Cl2	0.0368 (5)	0.0345 (5)	0.0597 (7)	−0.0025 (4)	−0.0048 (5)	−0.0173 (5)
Cl3	0.0496 (6)	0.0580 (7)	0.0504 (7)	−0.0108 (5)	−0.0205 (5)	0.0024 (5)
CS2	0.066 (7)	0.023 (4)	0.066 (7)	−0.003 (4)	0.004 (5)	−0.022 (4)
Cl4	0.0684 (17)	0.0590 (16)	0.0764 (19)	−0.0064 (14)	−0.0037 (15)	−0.0429 (15)
Cl5	0.0595 (18)	0.110 (3)	0.073 (2)	−0.010 (2)	−0.0058 (15)	−0.055 (2)

### Geometric parameters (Å, °)

**Table:**

Fe—N2	2.060 (3)	C41—H41A	0.9700
Fe—N4	2.066 (3)	C41—H41B	0.9700
Fe—N1	2.066 (2)	C51—C52	1.521 (5)
Fe—N3	2.067 (2)	C51—H51A	0.9700
Fe—Cl1	2.2430 (13)	C51—H51B	0.9700
N1—CA1	1.378 (4)	C61—C62	1.507 (5)
N1—CA2	1.383 (3)	C61—H61A	0.9700
N2—CA4	1.375 (4)	C61—H61B	0.9700
N2—CA3	1.376 (4)	C71—C72	1.516 (5)
N3—CA5	1.375 (4)	C71—H71A	0.9700
N3—CA6	1.383 (4)	C71—H71B	0.9700
N4—CA7	1.378 (4)	C81—C82	1.524 (5)
N4—CA8	1.378 (4)	C81—H81A	0.9700
CA1—CM1	1.388 (4)	C81—H81B	0.9700
CA1—CB1	1.447 (4)	C12—H12A	0.9600
CA2—CM2	1.381 (4)	C12—H12B	0.9600
CA2—CB2	1.443 (4)	C12—H12C	0.9600
CA3—CM2	1.382 (4)	C22—H22A	0.9600
CA3—CB3	1.448 (4)	C22—H22B	0.9600
CA4—CM3	1.386 (4)	C22—H22C	0.9600
CA4—CB4	1.440 (4)	C32—H32A	0.9600
CA5—CM3	1.388 (4)	C32—H32B	0.9600
CA5—CB5	1.444 (4)	C32—H32C	0.9600
CA6—CM4	1.385 (4)	C42—H42A	0.9600
CA6—CB6	1.443 (4)	C42—H42B	0.9600
CA7—CM4	1.390 (4)	C42—H42C	0.9600
CA7—CB7	1.449 (4)	C52—H52A	0.9600
CA8—CM1	1.387 (4)	C52—H52B	0.9600
CA8—CB8	1.437 (4)	C52—H52C	0.9600
CB1—CB2	1.366 (4)	C62—H62A	0.9600
CB1—C11	1.495 (4)	C62—H62B	0.9600
CB2—C21	1.504 (4)	C62—H62C	0.9600
CB3—CB4	1.371 (4)	C72—H72A	0.9600
CB3—C31	1.494 (4)	C72—H72B	0.9600
CB4—C41	1.493 (4)	C72—H72C	0.9600
CB5—CB6	1.360 (4)	C82—H82A	0.9600
CB5—C51	1.500 (4)	C82—H82B	0.9600
CB6—C61	1.497 (4)	C82—H82C	0.9600
CB7—CB8	1.359 (4)	CM1—HM1	0.9300
CB7—C71	1.501 (4)	CM2—HM2	0.9300
CB8—C81	1.498 (4)	CM3—HM3	0.9300
C11—C12	1.529 (5)	CM4—HM4	0.9300
C11—H11A	0.9700	CS1—Cl3	1.742 (4)
C11—H11B	0.9700	CS1—Cl2	1.749 (4)
C21—C22	1.525 (4)	CS1—HS1A	0.9700
C21—H21A	0.9700	CS1—HS1B	0.9700
C21—H21B	0.9700	CS2—Cl4	1.732 (10)
C31—C32	1.524 (4)	CS2—Cl5	1.798 (12)
C31—H31A	0.9700	CS2—HS2A	0.9700
C31—H31B	0.9700	CS2—HS2B	0.9700
C41—C42	1.521 (5)		
			
N2—Fe—N4	154.50 (10)	CB5—C51—H51A	109.2
N2—Fe—N1	87.08 (10)	C52—C51—H51A	109.2
N4—Fe—N1	86.96 (10)	CB5—C51—H51B	109.2
N2—Fe—N3	87.05 (10)	C52—C51—H51B	109.2
N4—Fe—N3	87.14 (10)	H51A—C51—H51B	107.9
N1—Fe—N3	153.12 (10)	CB6—C61—C62	113.0 (3)
N2—Fe—Cl1	102.63 (8)	CB6—C61—H61A	109.0
N4—Fe—Cl1	102.87 (8)	C62—C61—H61A	109.0
N1—Fe—Cl1	103.92 (7)	CB6—C61—H61B	109.0
N3—Fe—Cl1	102.96 (7)	C62—C61—H61B	109.0
CA1—N1—CA2	105.7 (2)	H61A—C61—H61B	107.8
CA1—N1—Fe	126.55 (18)	CB7—C71—C72	114.3 (3)
CA2—N1—Fe	126.22 (18)	CB7—C71—H71A	108.7
CA4—N2—CA3	105.6 (2)	C72—C71—H71A	108.7
CA4—N2—Fe	126.10 (19)	CB7—C71—H71B	108.7
CA3—N2—Fe	126.47 (19)	C72—C71—H71B	108.7
CA5—N3—CA6	105.4 (2)	H71A—C71—H71B	107.6
CA5—N3—Fe	126.67 (19)	CB8—C81—C82	111.8 (3)
CA6—N3—Fe	126.9 (2)	CB8—C81—H81A	109.3
CA7—N4—CA8	105.4 (2)	C82—C81—H81A	109.3
CA7—N4—Fe	126.25 (19)	CB8—C81—H81B	109.3
CA8—N4—Fe	126.63 (19)	C82—C81—H81B	109.3
N1—CA1—CM1	124.4 (3)	H81A—C81—H81B	107.9
N1—CA1—CB1	110.5 (2)	C11—C12—H12A	109.5
CM1—CA1—CB1	125.1 (3)	C11—C12—H12B	109.5
CM2—CA2—N1	125.2 (3)	H12A—C12—H12B	109.5
CM2—CA2—CB2	124.7 (3)	C11—C12—H12C	109.5
N1—CA2—CB2	110.1 (2)	H12A—C12—H12C	109.5
N2—CA3—CM2	125.1 (3)	H12B—C12—H12C	109.5
N2—CA3—CB3	110.7 (2)	C21—C22—H22A	109.5
CM2—CA3—CB3	124.2 (3)	C21—C22—H22B	109.5
N2—CA4—CM3	124.7 (3)	H22A—C22—H22B	109.5
N2—CA4—CB4	110.7 (3)	C21—C22—H22C	109.5
CM3—CA4—CB4	124.6 (3)	H22A—C22—H22C	109.5
N3—CA5—CM3	124.3 (3)	H22B—C22—H22C	109.5
N3—CA5—CB5	110.8 (3)	C31—C32—H32A	109.5
CM3—CA5—CB5	124.9 (3)	C31—C32—H32B	109.5
N3—CA6—CM4	124.7 (3)	H32A—C32—H32B	109.5
N3—CA6—CB6	110.2 (3)	C31—C32—H32C	109.5
CM4—CA6—CB6	125.1 (3)	H32A—C32—H32C	109.5
N4—CA7—CM4	124.7 (3)	H32B—C32—H32C	109.5
N4—CA7—CB7	110.4 (3)	C41—C42—H42A	109.5
CM4—CA7—CB7	124.9 (3)	C41—C42—H42B	109.5
N4—CA8—CM1	124.2 (3)	H42A—C42—H42B	109.5
N4—CA8—CB8	110.7 (3)	C41—C42—H42C	109.5
CM1—CA8—CB8	125.1 (3)	H42A—C42—H42C	109.5
CB2—CB1—CA1	106.5 (2)	H42B—C42—H42C	109.5
CB2—CB1—C11	128.5 (3)	C51—C52—H52A	109.5
CA1—CB1—C11	125.0 (3)	C51—C52—H52B	109.5
CB1—CB2—CA2	107.1 (2)	H52A—C52—H52B	109.5
CB1—CB2—C21	128.1 (3)	C51—C52—H52C	109.5
CA2—CB2—C21	124.7 (3)	H52A—C52—H52C	109.5
CB4—CB3—CA3	106.1 (2)	H52B—C52—H52C	109.5
CB4—CB3—C31	128.4 (3)	C61—C62—H62A	109.5
CA3—CB3—C31	125.5 (3)	C61—C62—H62B	109.5
CB3—CB4—CA4	106.9 (2)	H62A—C62—H62B	109.5
CB3—CB4—C41	127.5 (3)	C61—C62—H62C	109.5
CA4—CB4—C41	125.6 (3)	H62A—C62—H62C	109.5
CB6—CB5—CA5	106.5 (3)	H62B—C62—H62C	109.5
CB6—CB5—C51	128.7 (3)	C71—C72—H72A	109.5
CA5—CB5—C51	124.8 (3)	C71—C72—H72B	109.5
CB5—CB6—CA6	107.1 (3)	H72A—C72—H72B	109.5
CB5—CB6—C61	128.2 (3)	C71—C72—H72C	109.5
CA6—CB6—C61	124.5 (3)	H72A—C72—H72C	109.5
CB8—CB7—CA7	106.5 (3)	H72B—C72—H72C	109.5
CB8—CB7—C71	128.0 (3)	C81—C82—H82A	109.5
CA7—CB7—C71	125.5 (3)	C81—C82—H82B	109.5
CB7—CB8—CA8	107.0 (3)	H82A—C82—H82B	109.5
CB7—CB8—C81	128.1 (3)	C81—C82—H82C	109.5
CA8—CB8—C81	124.8 (3)	H82A—C82—H82C	109.5
CB1—C11—C12	112.0 (3)	H82B—C82—H82C	109.5
CB1—C11—H11A	109.2	CA8—CM1—CA1	126.6 (3)
C12—C11—H11A	109.2	CA8—CM1—HM1	116.7
CB1—C11—H11B	109.2	CA1—CM1—HM1	116.7
C12—C11—H11B	109.2	CA2—CM2—CA3	125.6 (3)
H11A—C11—H11B	107.9	CA2—CM2—HM2	117.2
CB2—C21—C22	112.0 (3)	CA3—CM2—HM2	117.2
CB2—C21—H21A	109.2	CA4—CM3—CA5	126.3 (3)
C22—C21—H21A	109.2	CA4—CM3—HM3	116.8
CB2—C21—H21B	109.2	CA5—CM3—HM3	116.8
C22—C21—H21B	109.2	CA6—CM4—CA7	126.0 (3)
H21A—C21—H21B	107.9	CA6—CM4—HM4	117.0
CB3—C31—C32	113.6 (2)	CA7—CM4—HM4	117.0
CB3—C31—H31A	108.9	Cl3—CS1—Cl2	112.1 (2)
C32—C31—H31A	108.9	Cl3—CS1—HS1A	109.2
CB3—C31—H31B	108.9	Cl2—CS1—HS1A	109.2
C32—C31—H31B	108.9	Cl3—CS1—HS1B	109.2
H31A—C31—H31B	107.7	Cl2—CS1—HS1B	109.2
CB4—C41—C42	112.3 (3)	HS1A—CS1—HS1B	107.9
CB4—C41—H41A	109.2	Cl4—CS2—Cl5	111.1 (6)
C42—C41—H41A	109.2	Cl4—CS2—HS2A	109.4
CB4—C41—H41B	109.2	Cl5—CS2—HS2A	109.4
C42—C41—H41B	109.2	Cl4—CS2—HS2B	109.4
H41A—C41—H41B	107.9	Cl5—CS2—HS2B	109.4
CB5—C51—C52	112.2 (3)	HS2A—CS2—HS2B	108.0
